# Lactate score classification of hepatocellular carcinoma helps identify patients with tumors that respond to immune checkpoint blockade therapy

**DOI:** 10.1007/s13402-023-00861-2

**Published:** 2023-08-23

**Authors:** Kai Jiang, Lili Zhu, Huizhen Huang, Liu Zheng, Zhuqing Wang, Xiaonan Kang

**Affiliations:** 1 Department of Biobank, Renji Hospital, Shanghai Jiao Tong University School of Medicine, Shanghai, 200127 China; 2 State Key Laboratory of Oncogenes and Related Genes, Shanghai Cancer Institute, Renji Hospital, Shanghai Jiao Tong University School of Medicine, Shanghai, 200127 China; 3 Department of Gastroenterology, Shanghai General Hospital, Nanjing Medical University, Shanghai, 201620 China

**Keywords:** Hepatocellular carcinoma, Lactate cluster, Lactate phenotype cluster, Lactate score, Immunotherapy

## Abstract

**Purpose:**

Hepatocellular carcinoma (HCC) responds poorly to immunotherapy, and the durable response rate is 10-20%. Here, we aim to characterize HCC classifications based on lactate genes to identify patients who may benefit from immunotherapy.

**Methods:**

Lactate-related genes were applied for HCC classification in the current study, and lactate Cluster 1 (LC1) and lactate Cluster 2 (LC2) were defined. Differential genes from LC1 and LC2 helped define the following lactate phenotype clusters: lactate phenotype Cluster 1 (LPC1), lactate phenotype Cluster 2 (LPC2) and lactate phenotype Cluster 3 (LPC3). Based on the cluster annotation, the lactate score was defined and analyzed to evaluate the immunotherapy response.

**Results:**

All the classified clusters were analyzed, and they showed different immune signatures. The survival rate of LPC3 was higher than that of LPC2 (LPC3 vs. LPC2, *P* = 0.027) and LPC1 (LPC3 vs. LPC1, *P* = 0.027). Then, the lactate score was annotated and confirmed to be effective in predicting responses to immune checkpoint blockade therapy.

**Conclusion:**

In the current study, we developed a classification system for HCC and defined the lactate score, which was validated to be partially effective in estimating responses among tumor patients.

**Supplementary Information:**

The online version contains supplementary material available at 10.1007/s13402-023-00861-2.

## Introduction

Lactate is primarily located in the tumor microenvironment. In most cancer cells, despite sufficient oxygen, glycolysis will be utilized for energetic accumulation. Excessive lactate will be formed, leading to the Warburg effect [1]. It has long been thought that lactate is the end product of the cell formation process and is a metabolic side product. In recent years, lactate has been reported to be a molecule with special biological functions. Lactate plays a crucial role as a carbon source and in transducing signals to receptor cells. A large body of literature indicates that lactate plays roles in modulating the tumor microenvironment, regulating escape from immune surveillance and influencing cancer cell proliferation, metastasis and tumor angiogenesis [2].

As an important metabolite of aerobic glycolysis, lactate is highly involved in tumor initiation and progression. During carcinogenesis, metabolic reprogramming is considered a hallmark of cancer [3]. In addition, metabolism process-related genes were utilized for hepatocellular carcinoma (HCC) classification.

Primary liver cancer ranks sixth in cancer incidence and is the third leading cause of cancer mortality worldwide. HCC comprises 75%-85% of liver cancer cases [4]. In recent years, advances have been made in elucidating the molecular pathogenesis of HCC, yet limited therapeutic options are currently available [5]. Growing numbers of reports have established various analytical approaches to classify HCC into multiple subtypes to expand the HCC therapeutic arsenal.

More recently, immune checkpoint blockade therapies have shown remarkable efficiency in solid cancer treatment. Such agents have also been introduced in HCC treatment, yet the overall response rate is only 10%-20% [6–8]. Thus, establishing a molecular classification of HCC, especially immune checkpoint blockade therapy-related classification, will help guide appropriate treatments that are suitable for patients. Herein, our lactate score from lactate phenotype clusters displays promising predictive value for immunotherapy response.

Intriguingly, HCCs were efficiently classified from a metabolic perspective, and 3 subclasses with active, intermediate and exhausted metabolic activities were proposed [9]. This implies the possibility that metabolite lactate might be used for HCC classification. In this study, we analyzed lactate-related genes and the lactate phenotype gene expression panel from 871 human HCC samples. Lactate-related gene data allowed us to identify lactate Cluster 1 (LC1) and lactate Cluster 2 (LC2) of HCC, which helped identify the lactate phenotype genes. Intriguingly, the following three HCC cohorts were also identified: lactate phenotype Cluster 1 (LPC1), lactate phenotype Cluster 2 (LPC2) and lactate phenotype Cluster 3 (LPC3). Based on lactate phenotype cluster involvement in HCC patient survival, clinical characterization and immune cell infiltration, the lactate score was defined as a promising approach to evaluate patient prognosis and response to immunotherapy.

## Materials and methods

### Selection of lactate-related genes

A total of 206 lactate-related genes were retrieved from previous literature [10–16], and the gene list is provided in Supplementary Table 1.

### Patients and samples

The gene expression profile and clinical data of HCC used in this study were obtained from The Cancer Genome Atlas (TCGA, http://cancergenome.nih.gov/), the International Cancer Genome Consortium (ICGC, www.icgc.org), and the Gene Expression Omnibus (GEO, http://www.ncbi.nlm.nih.gov/geo/). Of these, 418 samples from patients with HCC (368 tumor samples and 50 normal samples) from the TCGA-LIHC were used for the training cohort. A total of 232 and 221 HCC samples from the LIRI-JP cohort and GSE14520, respectively, served as the validation cohort. Three solid cancer cohorts (melanoma, urothelial cancer and gastric cancer cohorts) and a total of 159 diffuse large B cell lymphoma and Burkitt lymphoma samples from GSE4475 were utilized as an immunotherapy response evaluation.

### Clustering

The lactate-related gene distribution on chromatin was analyzed using the RCircos package in R. Principal component analysis (PCA) was used to confirm the ability to distinguish tumor and normal samples. Consensus clustering based on lactate-related gene expression was performed by the ConsensusClusterPlus package in R. The distance was based on pearson correlation, and the clustering method was PAM. One thousand repeated samples were carried out to ensure the stability of classification. This clustering led to the following 2 lactate clusters: lactate Cluster 1 (LC1) and lactate Cluster 2 (LC2). Then, differential analyses between lactate clusters were conducted by the DESeq2 package in R. Genes with absolute log2 FC > = 1 and adjusted *P* < 0.05 were selected, list of differential genes between lactate clusters is provided in Supplementary Table 2. Consensus clustering was performed based on the expression of the selected genes as described above. This clustering led to the following 3 lactate phenotype clusters: lactate phenotype Cluster 1 (LPC1), lactate phenotype Cluster 2 (LPC2) and lactate phenotype Cluster 3 (LPC3).

### Generation of lactate score and performance validation

A one-way Cox regression analysis was performed on lactate-phenotype genes. The genes were defined as significantly related to overall survival (OS) when *P* < 0.05, and dimensionality reduction was performed based on the prognosis-related genes. The lactate score was calculated by the following formula to construct a prognostic risk scoring model:

$$lactate\ score=\sum \left( PC{1}_i+ PC{2}_i\right)$$

The formula calculates the lactate score value for sample “i”.

The formula was also used in the validation set (GSE14520 and ICGC LIRI-JP) to calculate the risk score. The mid-standard values were applied to determine the optimal threshold for samples in the high-risk and low-risk groups. Kaplan–Meier survival analysis was used to assess the predictive power of the prognostic models.

### Correlation of lactate score with tumor hallmarks

To evaluate the relationship of the lactate score with tumor hallmarks, Spearman rank correlation analysis was performed. A hallmark with a *P* value <0.05 and an absolute corR >0.1 was referred to as associated with lactate score.

### Immunotherapy response prediction using lactate score

A total of 159 diffuse large B cell lymphoma and Burkitt lymphoma samples from GSE4475 were chosen for immunotherapy response evaluation. The other three cohorts (melanoma (*n* = 10), urothelial cancer (*n* = 22) and gastric cancer (*n* = 45) cohorts) were also used for the immunotherapy response evaluation. The percentage of complete response (CR)/partial response (PR) and progressive disease (PD)/stable disease (SD) patients was compared between the high-risk and low-risk lactate score groups. The lactate scores of CR/PR and PD/SD patients were also analyzed.

### Statistical analysis

The statistical computation in this study was analyzed with R programming. In the case of normally distributed variables, unpaired Student’s t test was used, while the Mann–Whitney U test was used in the case of nonnormally distributed variables. As parametric and nonparametric methods for comparing three groups, one-way analysis of variance and the Kruskal–Wallis tests of variance were used. A two-tailed *P* value of 0.05 was considered statistically significant. Chi-square tests or Fisher’s exact tests were used to analyze variables in the contingency tables.

## Results

### Characterizing the 206 lactate genes

**Fig. 1:**
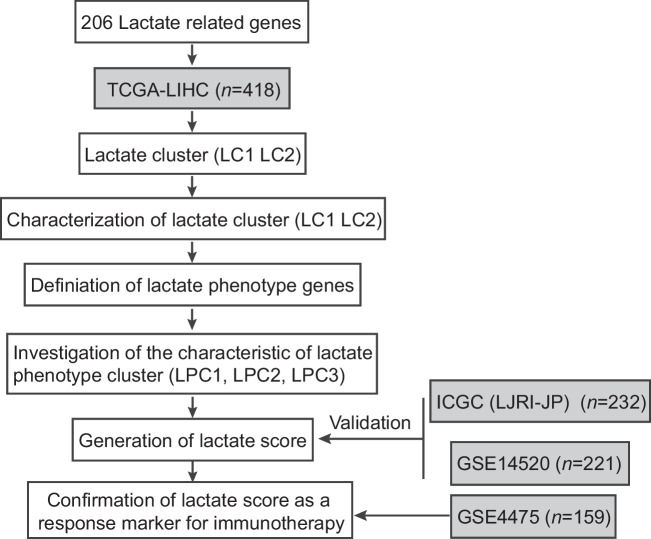
Study workflow. A total of 206 lactate-related genes were used for lactate cluster (LC1 and LC2) characterization. Differential expression analysis revealed lactate phenotype genes that led to the following 3 lactate phenotype clusters: LPC1, LPC2 and LPC3. The lactate score was defined based on the analysis of the survival-related lactate phenotype genes and was proven to be a response marker for immunotherapy. The training set was TCGA-LIHC (*n* = 418). The validation sets were ICGC (LIRI-JP) (*n* = 232) and GSE14250 (*n* = 221). The immunotherapy response confirmation set was GSE4475 (*n* = 159)

**Fig. 2:**
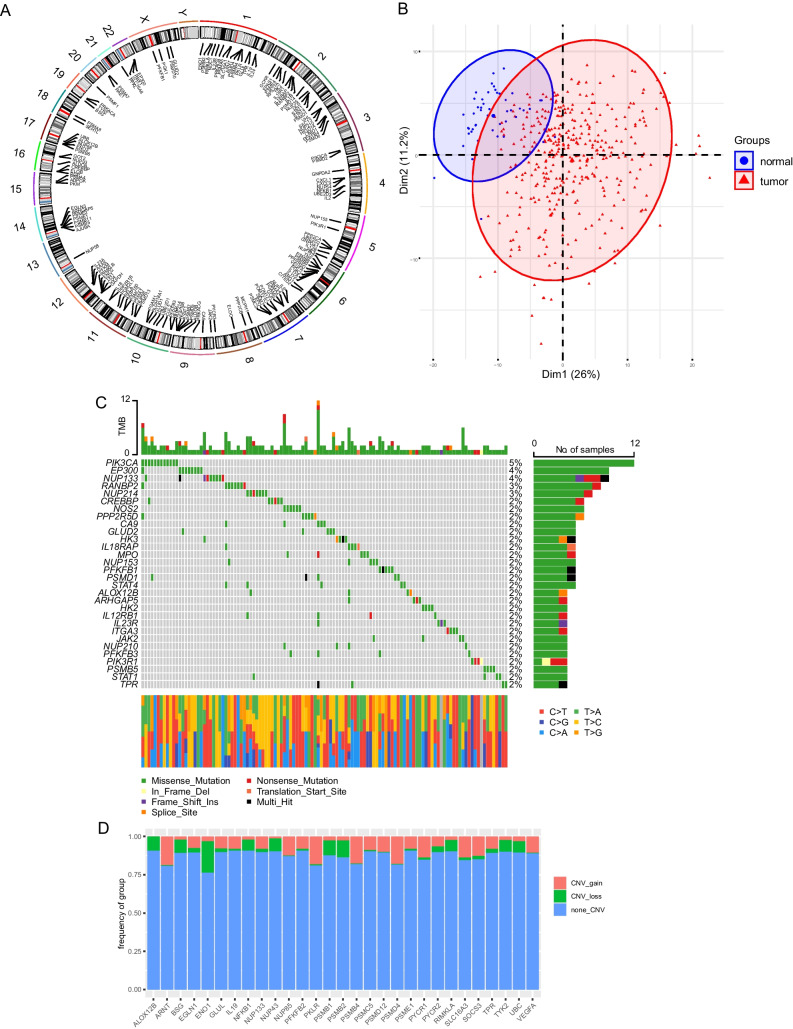
Characterization of lactate-related genes. **A**. The distribution of lactate-related genes on chromatin. **B**. PCA revealed the distinction of lactate-related genes in normal and tumor samples. **C**. The somatic mutation frequencies of lactate-related genes. **D**. The burden of somatic copy number aberrations of lactate-related genes

The flow chart delineating our systematic study is shown in Fig. 1. A total of 206 lactate-related genes were obtained by analyzing the lactate-related literature [10–16]. To better understand the lactate-related genes, we analyzed the gene distribution on chromatin (Fig. 2A). Almost all of the chromosomes contained at least 1 gene except chromosome Y. Notably, chromosome 2 had 15 lactate genes, including *GCKR, EPAS1, PSME4, RPS27A, HK2, IL18R1, IL18RAP, RANBP2, IL1B, PSMD14, NUP35, GLS, STAT1, STAT4* and *PSMD6*. There was only 1 gene, *NUP58,* on chromosome 13. To determine whether the 206 lactate genes could discriminate tumor and normal tissues, PCA was applied. The results revealed that HCC tissues were distinct from normal tissues (Fig. 2B). Recently, the tumoral genomic landscape has been linked to antitumor immunity. We next investigated the somatic mutation frequencies and the burden of somatic copy number variation of the lactate genes using the TCGA-LIHC dataset. The genes displaying high mutation frequency are visualized in Fig. 2C, including *PIK3CA* (5%), *EP300* (4%), *NUP133* (4%), *RANBP2* (3%), *NUP214* (3%), *CREBBP* (2%), *NOS2* (2%) and *PPP2R5D* (2%). The most common mutation type was missense mutation. The remaining mutated lactate genes were also involved in cancer progression. In terms of the copy number variation (Fig. 2D), some genes showed a high level of burden of gains, including *ARNT*, *PKLR*, *PSMD12*, *NUP85* and *VEGFA*. Some genes exhibited a high burden of losses, including *ENO1*, *PSMB2*, *PSMB1*, *BSG* and *ALOX12B*. Altogether, these data showed that the lactate genes were distributed over most chromosomes and harbored somatic mutations and copy number aberrations. These genes were mostly involved in cancer progression and therefore could be used for HCC classification.

### Defining lactate-related classes of HCC

**Fig. 3:**
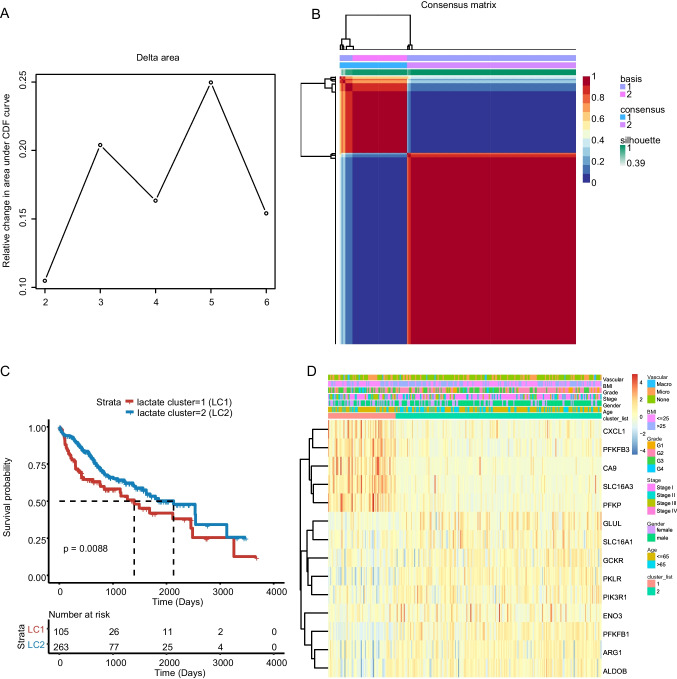
Identification of lactate clusters using consensus clustering. **A**. Consensus clustering using the 206 lactate-related genes. The cophenetic correlation coefficient for k = 2-6 is shown. **B**. The consensus matrix heatmap shown when k = 2. **C**. The survival of LC1 and LC2 in the TCGA-LIHC training set. The statistical analyses were performed by the log-rank test. **D**. The distribution of representative differentially expressed genes and clinicopathologic parameters (vascular invasion, body mass index (BMI), tumor grade, tumor stage, sex and age) between LC1 and LC2

A total of 368 tumor samples of patients with HCC from TCGA-LIHC were clustered based on their lactate gene expression profile. The groups were classified using the consensus clustering method. A comprehensive analysis of cophenetic correlation coefficients was conducted, and the optimal cluster number was chosen to be 2 (Fig. 3A). As shown in Fig. 3B, in the case of k = 2, the consensus matrix heatmap still maintains sharp and crisp boundaries, indicating robust and stable clustering of the samples. The results showed that HCC patients were well clustered into the 2 lactate clusters LC1 and LC2. We next explored the prognostic value of the two clusters. Patients in the LC2 group exhibited a longer median survival time (MST) (*n* = 263) than those in the LC1 group (*n* = 105) (*P* = 0.0088, Fig. 3C). Each class harbors its own lactate gene traits, with *CXCL1*, *PFKFB3*, *CA9,* etc.*,* highly expressed in LC1 and *GLUL*, *SLC16A1*, *GCKR,* etc.*,* overexpressed in LC2. The clinicopathologic parameter (vascular invasion, body mass index (BMI), tumor grade (*P* < 0.05), tumor stage (*P* < 0.05), sex (*P* < 0.05) and age) distributions between the 2 classes are also shown (Fig. 3D).

### Identifying lactate phenotype clusters of HCC

**Fig. 4:**
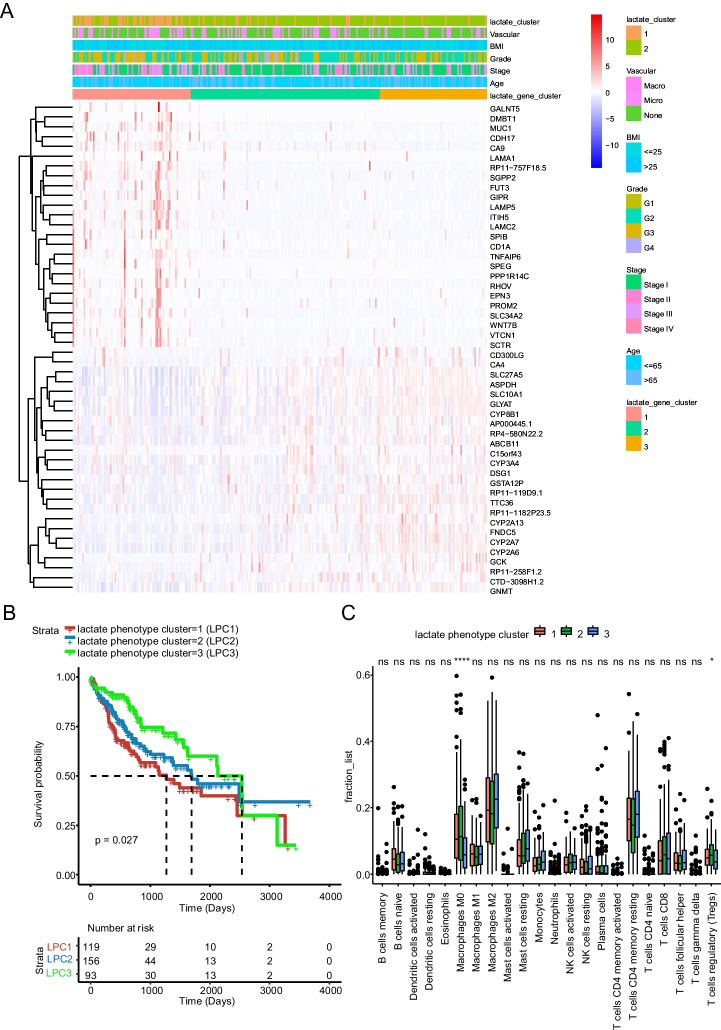
Characterization of lactate phenotype genes. **A**. The distribution of the representative differential lactate phenotype genes and the clinicopathologic parameters (vascular invasion, body mass index (BMI), tumor grade, tumor stage, sex and age) between LC1 and LC2 and between LPC1, LPC2 and LPC3. **B**. The survival of LPC1, LPC2 and LPC3 patients in the TCGA-LIHC training set. The statistical analyses were performed by the log-rank test. **C**. Boxplot of immune cell population abundance in LPC1, LPC2 and LPC3

To better characterize the lactate-involved phenotype application in HCC, we performed differential analyses comparing the lactate clusters. According to the threshold (the absolute log_2_ FC was > = 1 and the adjusted *P* was <0.05), 7036 genes were selected. These genes were named lactate phenotype genes. Consensus clustering of lactate phenotype genes was used to identify the following three subclasses: LPC1, LPC2 and LPC3. The differentially expressed lactate phenotype genes are visualized in Fig. 4A. The distributions of lactate cluster, vascular invasion, BMI, tumor grade, tumor stage and age are also displayed. To assess whether the newly identified lactate phenotype clusters had prognostic potential, we analyzed the survival of patients in each cluster. As predicted, patients belonging to LPC3 had a longer MST (*n* = 93) than those belonging to LPC2 (*n* = 156, *P* = 0.027) and LPC1 (*n* = 119, *P* = 0.027) (Fig. 4B). To our surprise, when analyzing the functional enrichment, immune-related processes were significantly enriched in biological process (BP). These processes included leukocyte-mediated immunity, human immune response and B cell receptor signaling pathway (Supplementary Fig. 1). Furthermore, the study revealed a decrease in the infiltration of both macrophage M0 and regulatory T cell (Treg) in LPC3 compared to LPC1 and LPC2, as depicted in Fig. 4C. These data indicated that lactate phenotype genes might be highly related to immune activity.

### Lactate score and performance validation

**Fig. 5:**
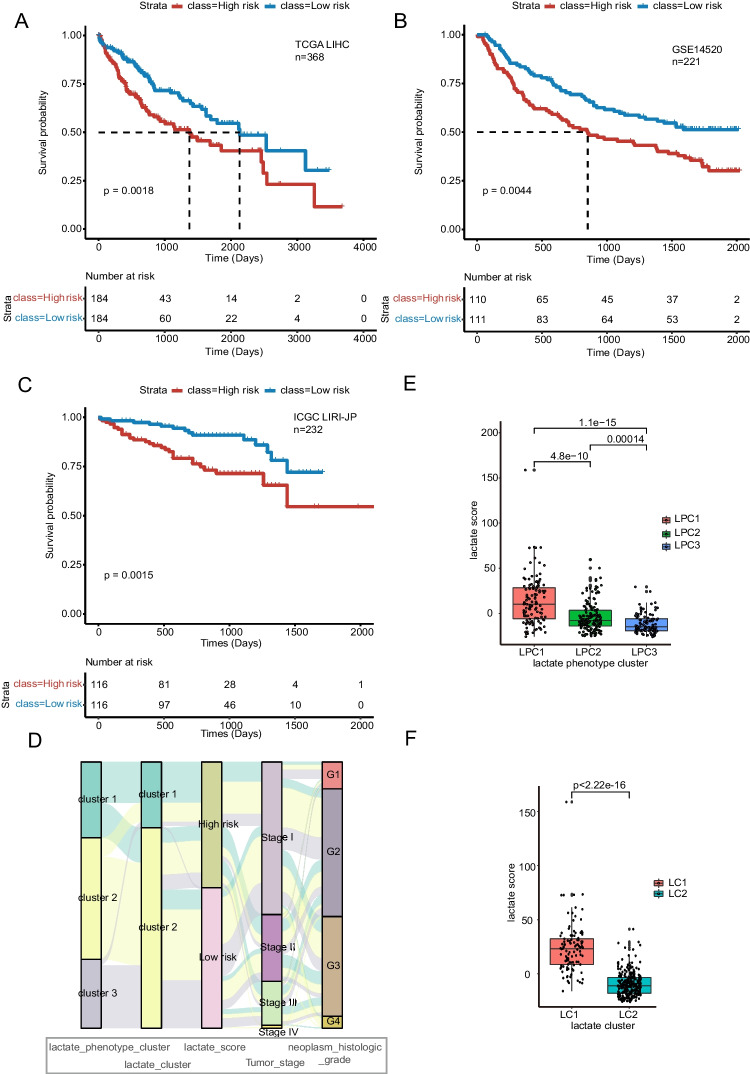
Definition of lactate score and performance validation. Survival differences between the lactate high-risk and low-risk groups are shown in the (**A**) TCGA-LIHC training set, (**B**) GSE14520 validation set and (**C**) ICGC (LIRI-JP) validation set. **D**. The distribution of high-risk and low-risk groups in lactate phenotype clusters, lactate clusters, tumor stage and neoplasm histologic grade shown in the Sankey diagram. **E**. The lactate score comparison of LPC1, LPC2 and LPC3. **F**. Lactate score comparison of LC1 and LC2. (****P < 0.0001)

We next sought to build a score for clinical application. Univariate Cox regression was performed, and 1507 marker genes related to survival were selected from lactate phenotype genes. We used these marker genes to score patients based on PCA. The score was referred to as the lactate score. Patients with lactate scores higher than the median score were defined as the high-risk group. Conversely, patients with lactate scores lower than the median score were defined as the low-risk group. Subsequently, the prognostic value of the lactate score was evaluated. Indeed, in the TCGA-LIHC testing dataset, patients in the low-risk group (*n* = 184) showed better survival than those in the high-risk group (*n* = 184, *P* = 0.0018) (Fig. 5A). Similarly, a significant prognostic difference was also observed in the GSE14520 validation dataset, with a longer MST for the low-risk group (*n* = 111) than for the high-risk group (*n* = 110, *P* = 0.0044) (Fig. 5B). In addition, similar results were also shown in the ICGC LIRI-JP validation dataset, and there was better prognostic value in the low-risk group (*n* = 116) than in the high-risk group (*n* = 116, P = 0.0015) (Fig. 5C).

We further explored the associations of the lactate score with the lactate cluster, lactate phenotype cluster, tumor stage and neoplasm histologic grade. The Sankey diagram (Fig. 5D) was delineated using the GGALLUVIAL R package. The results showed that most of the LPC3 patients were in the low-risk group, consistent with the data that both groups harbored good prognostic value. Similarly, the lactate score of LPC3 was lower than that of LPC2 (*P* = 0.00014) and LPC1 (*P* = 1.1e-15). Furthermore, LPC2 displayed a lower lactate score than LPC1 (*P* = 4.8e-10) (Fig. 5E), in accordance with its good survival performance. Interestingly, LC2 also exhibited a lower lactate score than LC1 (*P* < 2.22e-16) (Fig. 5F), as with the prognostic comparison between the two clusters. All these data showed that clusters with good prognostic performance maintained low lactate scores, which indicates that the lactate score has strong value for clinical applications.

### Correlation of lactate score with tumor hallmark and immune signature

**Fig. 6:**
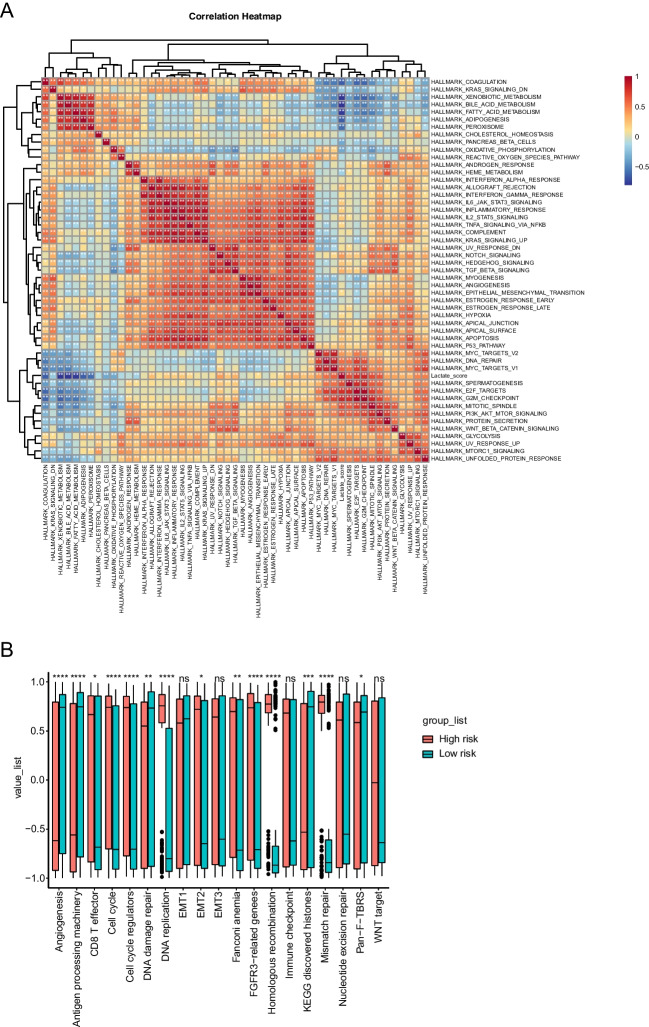
Delineation of the correlation of the lactate score with tumor hallmarks and the immune signature. **A**. Heatmap describing the relationship between the lactate score and tumor hallmarks. **B**. Immune signature differences between the high-risk and low-risk groups. (*P < 0.05, **P < 0.01, ***P < 0.001, ****P < 0.0001)

To assess the relationship of the lactate score with tumor hallmarks, Spearman rank correlation analysis was performed. The hallmark was associated with lactate score if the *P* value was <0.05 and the absolute corR >0.1. Most of the hallmarks were related to the lactate score, as shown in Fig. 6A. Tumor-specific hallmarks such as hypoxia, the P53 pathway, MYC targets, PI3K-AKT-mTOR signaling and WNT-beta-CATENIN signaling were related to the lactate score. In addition, immune response hallmarks such as interferon alpha/gamma response, inflammatory response, IL6-JAK-STAT3 signaling and IL2-STAT5 signaling were associated with lactate score. These data indicated that the lactate score can be applied for the evaluation of both tumor status and immune response condition for the patient. To further explore the effect of the lactate score on the immune signature, we analyzed the immune signature difference between the high-risk and low-risk groups. The immune signature was deciphered with the ssGESA algorithm and visualized in Fig. 6B. In accordance with the immune hallmarks, some of the immune signatures were significantly different between the two groups. Notably, M0 macrophages, naïve CD4 T cells, follicular helper T cells, gamma delta T cells and regulatory T cells (Tregs) scored higher in the high-risk group than in the low-risk group. Resting mast cells, monocytes and resting NK cells were scored higher in the low-risk group than in the high-risk group. Altogether, these data showed that the immune signature between the two groups was different, indicating that the infiltration of immune cells was associated with the level of lactate in the microenvironment.

### Survival and sensitivity to immunotherapy were distinct between the high/low-risk groups

**Fig. 7:**
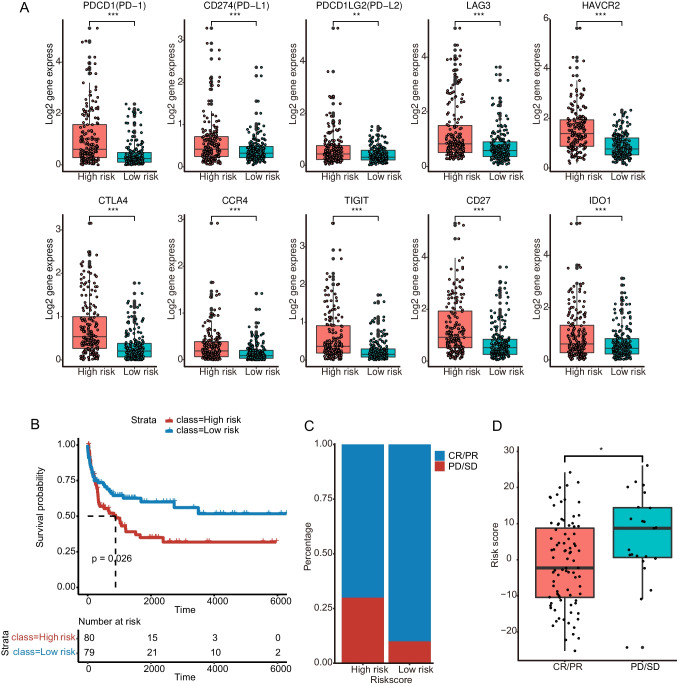
Distinct survival and sensitivity to immunotherapy between the high-risk and low-risk groups. **A**. The immune checkpoint marker expression differences between the high-risk and low-risk groups. The statistical significance was determined by the Wilcoxon rank-sum test. **B**. The survival of high-risk and low-risk groups in the GSE4475 set. **C**. The ratio of CR/PR and PD/SD patients in the high-risk and low-risk groups. **D**. Lactate score comparison between CR/PR and PD/SD patients. (*P < 0.05)

Considering the profound differences in immune-related pathways between the high-risk and low-risk groups, we wondered if the immunotherapy response was different between the two groups. The expression panel of immune checkpoint genes showed that the low-risk group had significantly lower expression levels. These genes included *PDCD1* (PD-1), *CD274* (PD-L1), *PDCD1LG2* (PD-L2), *LAG3*, *HAVCR2*, *CTLA4*, *CCR4*, *TIGIT*, *CD27* and *IDO1* (Fig. 7A). There were no public HCC immunotherapy cohorts with transcriptomic data available during this period. Other cohorts were used to explore whether the lactate score could serve as a response marker for immunotherapy. We analyzed immunotherapy datasets from cohorts of melanoma [17], urothelial cancer [18], gastric cancer [19] and the GEO dataset GSE4475 for validation. As depicted in Fig. 7C of the GSE4475 data, in the low-risk group, the percentage of complete response (CR)/partial response (PR) patients was 90%, and the remaining 10% had progressive disease (PD)/stable disease (SD). Nevertheless, in the high-risk group, the percentage of CR/PR patients was 69%, and the remaining 31% had PD/SD. This is consistent with the lower risk score in the CR/PR patients than in the PD/SD patients (*P* = 0.011) (Fig. 7D). In particular, the survival of the low-risk group was also better than that of the high-risk group in the GSE4475 dataset (Fig. 7B), as with other datasets previously mentioned. The data from the three solid cancer cohorts indicated that the percentages of CR/PR patients in the low-risk group were higher than those in the high-risk groups (Supplementary Fig. 2A, C, E). No significant differences were shown between the CR/PR and PD/SD groups in risk score, yet the median values of the risk score of the CR/PR groups were lower than those of the PD/SD groups (Supplementary Fig. 2B, D, F). This might be due to the patient numbers in the cohorts were not large enough or due to the total CR/PR ratio (melanoma cohort 6/10, urothelial cancer cohort 7/22, gastric cancer cohort 12/45) in the cohorts is low. Although the difference is not significant, the median values difference indicated the lactate score might be helpful in immunotherapy response prediction. Altogether, these results revealed that patients with low lactate scores were more likely to respond to immunotherapy. Thus, the lactate score could be used as a candidate marker to evaluate immunotherapy response.

## Discussion

Recently, immune checkpoint blockade therapy has shown remarkable efficiency in many types of solid cancers and has revolutionized the field of cancer treatment [5]. Clinically, such therapy has also been used for first- and second-line HCC treatment. However, not all patients respond to such therapy. In terms of HCC, only a modest fraction of HCC patients can benefit from immunotherapy treatment, with a durable response rate of 10-20% [6–8]. Therefore, there is an urgent need to define new methods that accurately predict responses to immune checkpoint blockade therapy [20]. Currently, accumulating efforts have been put into predicting patients that would respond to such kinds of immunotherapy. In an immune-related analysis, 2 subclasses of HCCs were identified with adaptive and exhausted immune responses, some of which were susceptible to immune checkpoint blockade therapies [5]. Furthermore, the same group further delineated the immunogenomic classification of HCC based on their previous study to predict responses or resistance to immunotherapy [20]. From the metabolic perspective, Yang et al. identified an HCC subclass that exhibited high sensitivity to immune checkpoint blockade therapy [9]. However, the response rate remains low. Here, we tried to offer a new method to predict true responders from the lactate perspective.

Lactate had long been recognized as a waste product of aerobic glycolysis until recently. It was reported that lactate plays a vital role in affecting the response to checkpoint therapy in tumors [21]. Thus, it is possible to define HCC from the lactate perspective. In our study, lactate-related genes were selected and applied for the classification of HCC into the following lactate clusters: LC1 and LC2. Based on the differential analyses between LC1 and LC2, another 3 clusters were identified as follows: LPC1, LPC2 and LPC3. The survival time of the patients in the 3 clusters were significantly different, with longer survival observed in LPC3 patients than in LPC1 and LPC2 patients. Moreover, immune-related processes were significantly enriched in the lactate phenotype clusters. In addition, immune cell infiltration (macrophage M0 and T regular cells) was also lower in LPC3 patients than in LPC1 and LPC2 patients, indicating that these clusters were related to immune activity. Thus, for the clinical use of the lactate phenotype, the lactate score was defined and delineated. The lactate score was shown to accurately predict responders to immune checkpoint blockade therapy.

Lactate has been reported to be involved in tumor progression. Increased aerobic glycolysis is a hallmark of cancer. Lactate was indicated to rewire the tumor microenvironment and power tumor malignancy [2]. Moreover, glucose uptake was reported to be correlated with poor prognosis in cancer patients [22]. Among patients treated with anti-PD-1, high levels of lactate dehydrogenase (LDH), a key enzyme in pyruvate conversion into lactate, have also been linked to a poor prognosis and outcome [21, 23–25]. In addition, lactate concentration was confirmed to be positively related to the incidence of tumor metastasis and reduced survival time [26–28]. Altogether, these data showed that lactate is of pivotal value in evaluating the prognosis of cancer patients. Thus, in the current study, 206 lactate genes were selected and delineated. Among them, there were genes with a high mutation frequency, including *PIK3CA* (5%) and *EP300* (4%). Mutations in *PIK3CA* are among the most frequent in a number of cancer types [29–31]. PI3K signaling blockade leads to the inhibition of glycolysis in tumors, including inhibition of the production of the downstream product of lactate [32]. *EP300* encodes the histone acetyltransferase p300, which is a tumor suppressor [33]. Mutation of *EP300* mediates Wnt/β-catenin–independent tumor growth [34]. Additionally, *EP300* is a coactivator of hypoxia-induced Factor 1 alpha (*HIF1A*), which stimulates hypoxia-induced genes such as *VEGF* [35]. Intriguingly, immune-related processes were significantly enriched in the BP category among the 3 LPC clusters. Specifically, macrophage M0 and Treg infiltration were shown to be lower in LPC3 than in LPC1 and LPC2. This is consistent with the results in the immune signature delineation of the lactate score, and cell infiltration was lower in the low-risk lactate score group than in the high-risk group. Tumor-associated macrophages (TAMs) preferentially accumulated in hypoxic tumors [36], where lactate is always abundant [2]. Moreover, Tregs are also adapted to the lactate-rich microenvironment [37]. Thus, our data were in line with that of previous studies.

For better application of the lactate phenotype, we defined the lactate score. It is easily used and partially effective in predicting responses to immune checkpoint blockade therapy. The low-risk group showed better survival than the high-risk group in both the testing dataset (TCGA-LIHC) and the validation datasets (GSE14520 and ICGC LIRI-JP). This is in accordance with the finding that a high level of lactate is correlated with poor survival [38]. Immune signature analysis showed that the high-risk group had high levels of M0 macrophages, naïve CD4 T cells, follicular helper T cells, gamma delta T cells and regulatory T cells (Tregs). The low-risk group had high levels of resting mast cells, monocytes and resting NK cells. It has been reported that high infiltration of M0 macrophages [39], naïve CD4 T cells [40], gamma delta T cells [41, 42] and regulatory T cells (Tregs) [40] is correlated with poor prognosis in cancer patients. Conversely, high levels of mast cells [43] and NK cells [44] were reported to be associated with favorable survival. These previous data were consistent with the good prognosis of patients in the low-risk group. Furthermore, Tregs suppress antitumor immunity, hampering effective antitumor immune responses [45]. NK cells serve as tumor immunosurveillance cells and can regulate T cell infiltration into the tumor [44]. Low levels of Tregs and high levels of NK cells in the low-risk group indicated a favorable microenvironment for immunotherapy. All 10 immune checkpoint genes analyzed here were lower in the low-risk group. According to the GSE4475 immunotherapy data analyzed, the low-risk group showed a higher level of CR/PR and a lower level of PD/SD than the high-risk group. The lactate score of the PD/SD patients was significantly higher than that of the CR/PR patients. Yet, this is not the case in all kinds of tumors. In this respect, the low-risk group displayed partially better response to immunotherapy. Therefore, a low lactate score could be used as an indicator for response to immunotherapy.

In summary, lactate-related genes were applied for HCC classification (LC1 and LC2) in the current study. Differential genes from LC1 and LC2 helped define the following lactate phenotype clusters: LPC1, LPC2 and LPC3. All the classified clusters were analyzed, and they had different survival rates (LC1 vs. LC2, LPC1 vs. LPC2 vs. LPC3) and immune signatures (LC1 vs. LC2, LPC1 vs. LPC2 vs. LPC3). Then, the lactate score was annotated and confirmed to be partially effective in predicting responses to immune checkpoint blockade therapy. Further studies validating lactate score application are needed in a larger cohort of patients treated with immune checkpoint blockade therapy.

## Limitations of the study

In this study we developed the lactate score serves as a valuable predictor for the prognosis of HCC patients, meanwhile the lactate score was evaluated as a candidate predictor for immunotherapeutic response. Notably, there is a limitation in the immunotherapeutic response prediction. Cohorts from melanoma, urothelial cancer, gastric cancer and the diffuse large B cell lymphoma and Burkitt lymphoma (GSE4475) cohorts were used for evaluating the immunotherapy response. While the differences from the first three cohorts were not significant and only GSE4475 dataset showed significant differences in the immunotherapy prediction data. This indicates the lactate score can be partially helpful in predicting the responders to immunotherapy. More immunotherapy cohorts especially HCC immunotherapy cohorts are needed for evaluating the predictive power for immunotherapeutic response of lactate score.

### Supplementary Information

ESM 1 (PDF 401 kb)

ESM 2 (PDF 426 kb)

ESM 3 (XLS 5 kb)

ESM 4 (XLSX 415 kb)

## Data Availability

All the data on which the conclusions are based are either in the main manuscript or its additional files.
