# Supplementary material for: Lactate score classification of hepatocellular carcinoma helps identify patients with tumors that respond to immune checkpoint blockade therapy
Source: Cell Oncol (Dordr). 2023 Aug 23;47(1):175–88. doi: 10.1007/s13402-023-00861-2 (PMC10899304; doi:10.1007/s13402-023-00861-2)

Supplementary Fig. 1. The top 10 processes enriched in the biological process (BP) of the lactate phenotype clusters.

A

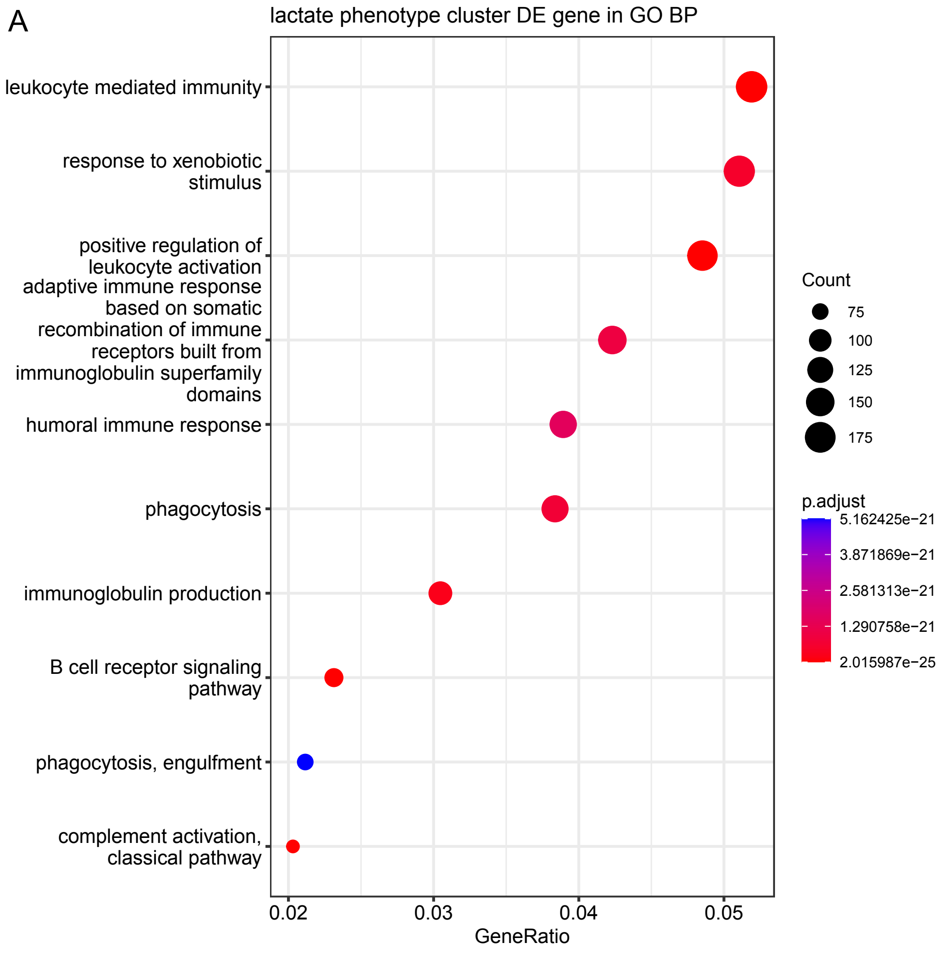

Supplement: Supplementary file 1 — (PDF 401 kb) [file 13402_2023_861_MOESM1_ESM.pdf]
