# Supplementary material for: Lactate score classification of hepatocellular carcinoma helps identify patients with tumors that respond to immune checkpoint blockade therapy
Source: Cell Oncol (Dordr). 2023 Aug 23;47(1):175–88. doi: 10.1007/s13402-023-00861-2 (PMC10899304; doi:10.1007/s13402-023-00861-2)

Supplementary Fig. 2. Sensitivity to immunotherapy between the high-risk and low-risk groups in melanoma (A, B), urothelial cancer (C, D) and gastric cancer (E, F) cohorts. The ratio of CR/PR and PD/SD patients in the high-risk and low-risk groups (A, C, E). Lactate score comparison between CR/PR and PD/SD patients (B, D, F).

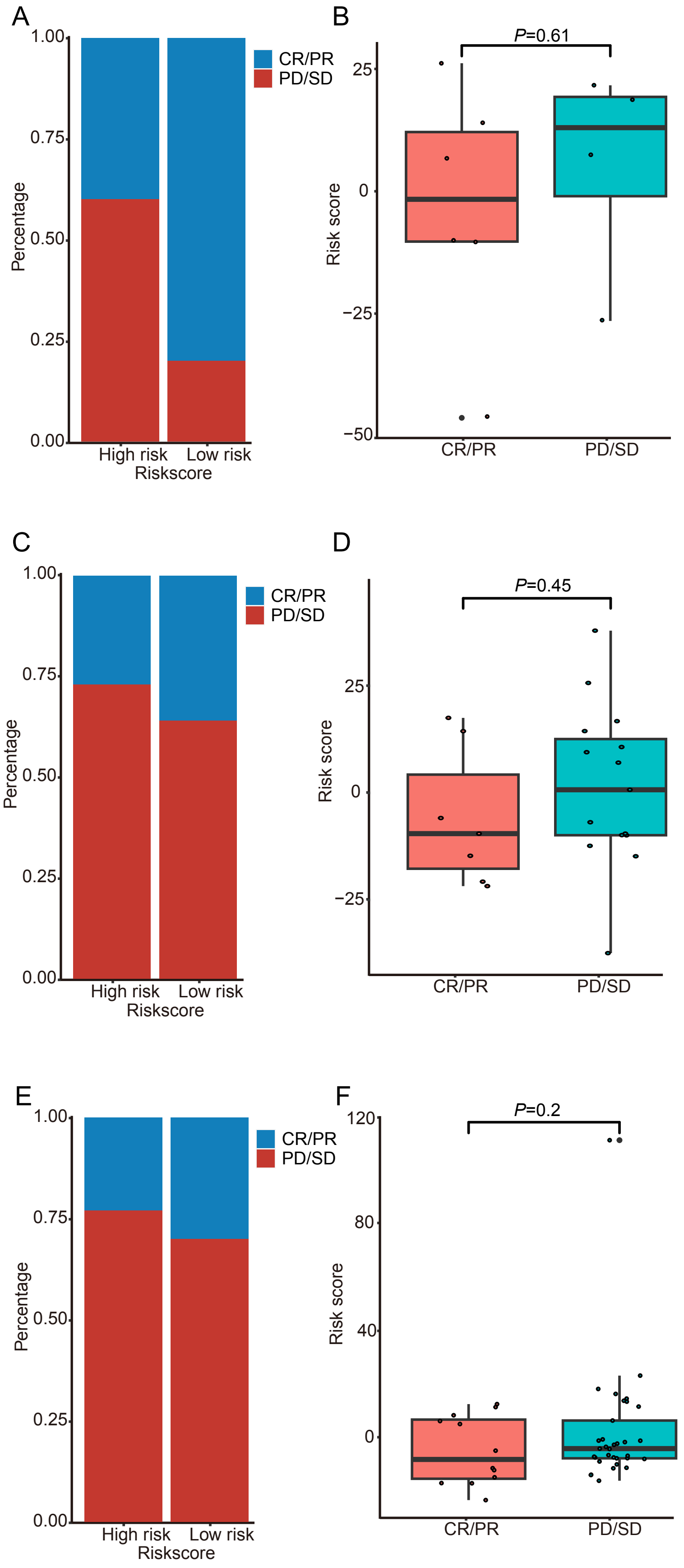

Supplement: Supplementary file 2 — (PDF 426 kb) [file 13402_2023_861_MOESM2_ESM.pdf]
